# Movements, Home-Range Size and Habitat Selection of Mallards during Autumn Migration

**DOI:** 10.1371/journal.pone.0100764

**Published:** 2014-06-27

**Authors:** Daniel Bengtsson, Alexis Avril, Gunnar Gunnarsson, Johan Elmberg, Pär Söderquist, Gabriel Norevik, Conny Tolf, Kamran Safi, Wolfgang Fiedler, Martin Wikelski, Björn Olsen, Jonas Waldenström

**Affiliations:** 1 Centre for Ecology and Evolution in Microbial Model Systems, Linnaeus University, Kalmar, Sweden; 2 Division of Natural Sciences, Kristianstad University, Kristianstad, Sweden; 3 Ottenby Bird Observatory, Ottenby 401, Degerhamn, Sweden; 4 Max Planck Institute for Ornithology, Dept. of Migration and Immuno-Ecology, Am Obstberg 1, Radolfzell, Germany and University of Konstanz, Dept. of Biology, Konstanz, Germany; 5 Section of Infectious Diseases, Department of Medical Sciences, Uppsala University, Uppsala, Sweden; Institute of Ecology, Germany

## Abstract

The mallard (*Anas platyrhynchos*) is a focal species in game management, epidemiology and ornithology, but comparably little research has focused on the ecology of the migration seasons. We studied habitat use, time-budgets, home-range sizes, habitat selection, and movements based on spatial data collected with GPS devices attached to wild mallards trapped at an autumn stopover site in the Northwest European flyway. Sixteen individuals (13 males, 3 females) were followed for 15–38 days in October to December 2010. Forty-nine percent (SD = 8.4%) of the ducks' total time, and 85% of the day-time (SD = 28.3%), was spent at sheltered reefs and bays on the coast. Two ducks used ponds, rather than coast, as day-roosts instead. Mallards spent most of the night (76% of total time, SD = 15.8%) on wetlands, mainly on alvar steppe, or in various flooded areas (e.g. coastal meadows). Crop fields with maize were also selectively utilized. Movements between roosting and foraging areas mainly took place at dawn and dusk, and the home-ranges observed in our study are among the largest ever documented for mallards (mean  = 6,859 ha; SD = 5,872 ha). This study provides insights into relatively unknown aspects of mallard ecology. The fact that autumn-staging migratory mallards have a well-developed diel activity pattern tightly linked to the use of specific habitats has implications for wetland management, hunting and conservation, as well as for the epidemiology of diseases shared between wildlife and domestic animals.

## Introduction

The mallard (*Anas platyrhynchos*) is the most numerous and widespread dabbling duck in the world and an important quarry species in the northern hemisphere. It is often used as a model species for research on ecological processes [1], harvest management [2], and disease [3]–[5]. In Europe, Asia and North America, most mallard populations are migratory and depend on suitable stopover sites along the route to complete their annual migrations [6], [7]. However, stopover ecology is one of the least studied aspects of avian migration [8], and even for a well-studied species like the mallard, little has been published about habitat selection, time-budgets, foraging behaviour, and diet selection during migration. Although food resources of the widely sympatric Eurasian teal (*Anas crecca crecca*) have been investigated at the flyway level [9], [10], detailed data on an individual basis are very scarce for migrating dabbling ducks. Moreover, in an extensive review of North American dabbling ducks, Callicutt *et al.* (2011) concluded that there were no studies of food use at the scale of appropriate daily home-range sizes [11].

Active migration requires energy, which birds build up as fat reserves before departing [12], and most migrating birds depend on refuelling along the route [13], [14]. The overall speed of migration is therefore determined by spatial availability of stopover sites and refuelling rates within these sites [12], [13], [15], [16]. Based on analyses of oesophagus and gut contents, mallards generally shift from an invertebrate-dominated diet during spring and summer to a largely granivorous diet during autumn and winter [17]–[22]. Apart from seeds and various water plants, mallards feed on agricultural crops at this time of the year. Accordingly, ducks may aggregate in major grain-growing regions in autumn and winter [16], sometimes causing extensive crop damage [23]. Diet selection depends on food availability, and crop choice by farmers could influence stopover behaviours and spatial dynamics in ducks [24].

Mallards use separate habitats for roosting and foraging, at least in winter [25]–[28]. In general terms, the daily home-range is thought to primarily depend on the spatial distribution of food resources [29], but it may also be affected by predation and hunting pressure, disturbance, and inter- and intraspecific population densities [30]. Hence, home-range sizes vary among regions [31], but also between seasons. For practical reasons, most studies on dabbling ducks have been carried out in daylight [32]. This is an obvious shortcoming with respect to foraging behaviour, as mallards forage mainly at night for a large part of the year [26], [33], [34].

Modern technology provides new opportunities to locate birds without visually following them. By utilizing small, portable GPS devices, birds can be tracked and highly accurate spatial data can be collected remotely. In the present study, we equipped autumn-staging mallards in SE Sweden with local read-out GPS transmitters and followed their movements in a coastal agricultural landscape. The aim was to better understand patterns of habitat use, local movements, home-range sizes, and habitat selection of individual mallards at an important stopover site in the Northwest European flyway [35].

## Methods

### Ethics statement

This research was carried out following strict protocols during all steps that included contacts with the birds under study. Ethical approval for trapping, sampling, and keeping birds was obtained from the Swedish Animal Research Ethics Board (“Linköpings djurförsöksetiska nämnd”, reference number 46–09). Birds were handled for the minimum amount of time possible. There was no evidence that any individuals died, were hampered, or otherwise negatively affected as a direct result of having a GPS tag attached.

### Study area

Öland is a Swedish island in the southern Baltic Sea. It is approximately 140 km from N to S and 18 km E to W at the widest. Öland is a major stopover site for dabbling ducks during autumn, due to a rich food supply and its location between breeding and wintering areas [36]–[38]. Most of the coast consists of shallow, stony beaches, many clad with seaweed beds. Reefs and wave-protected bays are rare. The southern part of Öland is dominated by farmland, pastures and alvar steppe. The alvar is a limestone-rich steppe habitat which, in Sweden, is limited mainly to the Baltic Sea islands of Öland and Gotland. It is an open and flat habitat with little or no soil. The scrubby vegetation is dominated by grass and juniper, and is often grazed by cattle. There are only a few permanent, small wetlands on the southern part of the island, but due to rains the alvar habitat contains several ephemeral wetlands in late autumn.

### Trapping

Ottenby Bird Observatory (56°12′N, 16°24′E) uses a stationary trap specifically designed for catching ducks for banding and epidemiological studies (e.g. sampling for avian influenza viruses). The trap is oblong with several funnel-shaped entrances on one side, through which ducks are attracted by bait grain. The trap is cleared daily, at which time ducks that have entered it are herded into a separate section where they are caught, placed in individual cardboard boxes, and taken to a nearby field lab (see [5] for further details). Sampling for this study started on 25 October 2010, during the peak of mallard autumn migration.

### GPS devices

After being banded and measured, 40 wild mallards (24 males and 16 females, all first calendar year birds), caught 25–27 October, were equipped with a GPS device and then released at the trap. The devices were of the type “Bird 2AA2” (e-obs Digital Telemetry, Grünwald, Germany) with the maximum measurement 90×30×16 mm, and were equipped with a 70 mm long antenna, angled backwards. Total device weight was 51 grams (approximately 5% of mallard body mass). The device was attached with a harness (see [39] for further details) and a 4 mm thick neoprene pad glued to the bottom of the device. Each device was set to record one data point every 15 minutes, including information about time, location (usually accurate to within 10 meters), and speed over ground. The expected battery life was estimated to be three weeks minimum; some of the devices were still functioning after almost seven weeks.

### Fieldwork

The birds were tracked on a daily basis using portable download equipment with receiver. Under ideal conditions, standing at the top of the lighthouse at the southern tip of Öland, 40 m above ground level, data could be downloaded from a distance of 4 km. This was done every day. From the ground, however, downloading data was sometimes difficult even within a few hundred metres. To ensure that we collected data from all tagged ducks remaining in the study area, we almost daily flew over southern Öland with a light airplane on which a receiver was mounted. On three flying occasions, we also searched the northern part of the island and the mainland coast, without finding any tagged individuals. On days with unsuitable weather for airplane flights (>50% of days), 25–30 km of the east coast, as well as a bay on the west coast of the island where birds had previously been located, were covered by car and foot. After 17 November, the airplane was used only once (8 December) due to poor weather. Instead, efforts on the ground were intensified. We were able to download data from all but two devices, yielding a gross sample from 38 birds. Field work was terminated after 47 days. At that time, at least one duck was still staging on southeast Öland.

### Analysed data

We excluded birds that showed movement patterns that suggested they were habituated to the duck trap, as these birds' movements were predominately driven by the predictable food source at the trap. Some mallards frequented the Ottenby area and the duck trap during the first week after the tracking device was attached, then abruptly and simultaneously left for another day-roost and started visiting crop fields and/or inland wetlands. Ducks were considered to behave naturally (i.e. not showing trap-dependence) 24 hours after visiting the trap area (<20 metres from the trap), if never returning thereafter. This left us with data from 16 ducks (13 males, 3 females). For these individuals, data on natural behaviour (as described above) were available for on average 24 days (range: 15–38). Some GPS fixes were missing, but in general data could be considered continuous, as average time lag between consecutive locations was only slightly more than the programmed 15 minutes (Table 1). GPS data for the 16 mallards were analysed with respect to habitat use (data points attributed to each habitat), habitat preference, home-range size, and distance of movements. Data points assumed to be from ducks in flight (i.e. if speed was >4 m/s) were excluded. The mean number of data points per duck was 2,180 (min = 1,330; max = 3,366; SD = 646). All the GPS data is stored at Movebank (www.movebank.org), and accessible under the project name “Mallard Waldenstrom Ottenby Sweden” (Movebank ID 3109235).

**Table 1:** Average time lag (in minutes) between consecutive fixes for 16 mallards studied on southern Öland, Sweden, October – December 2010.

Individual	Average time lag between consecutive fixes (min)	Standard error
1425	15.7	13.7
1426	15.4	2.5
1427	15.5	2.9
1429	15.3	2.5
1440	15.3	2.4
1441	15.1	1.5
1445	15.3	2.1
1451	15.3	2.3
1452	15.4	2.9
1456	15.4	2.9
1457	15.4	2.8
1459	15.3	2.2
1460	15.4	2.6
1461	15.3	2.5
1462	15.7	12.6
1463	15.4	2.7
Mean all	15.4	3.83
StDev	0.14	3.66

### Habitat definitions

**Figure 1:**
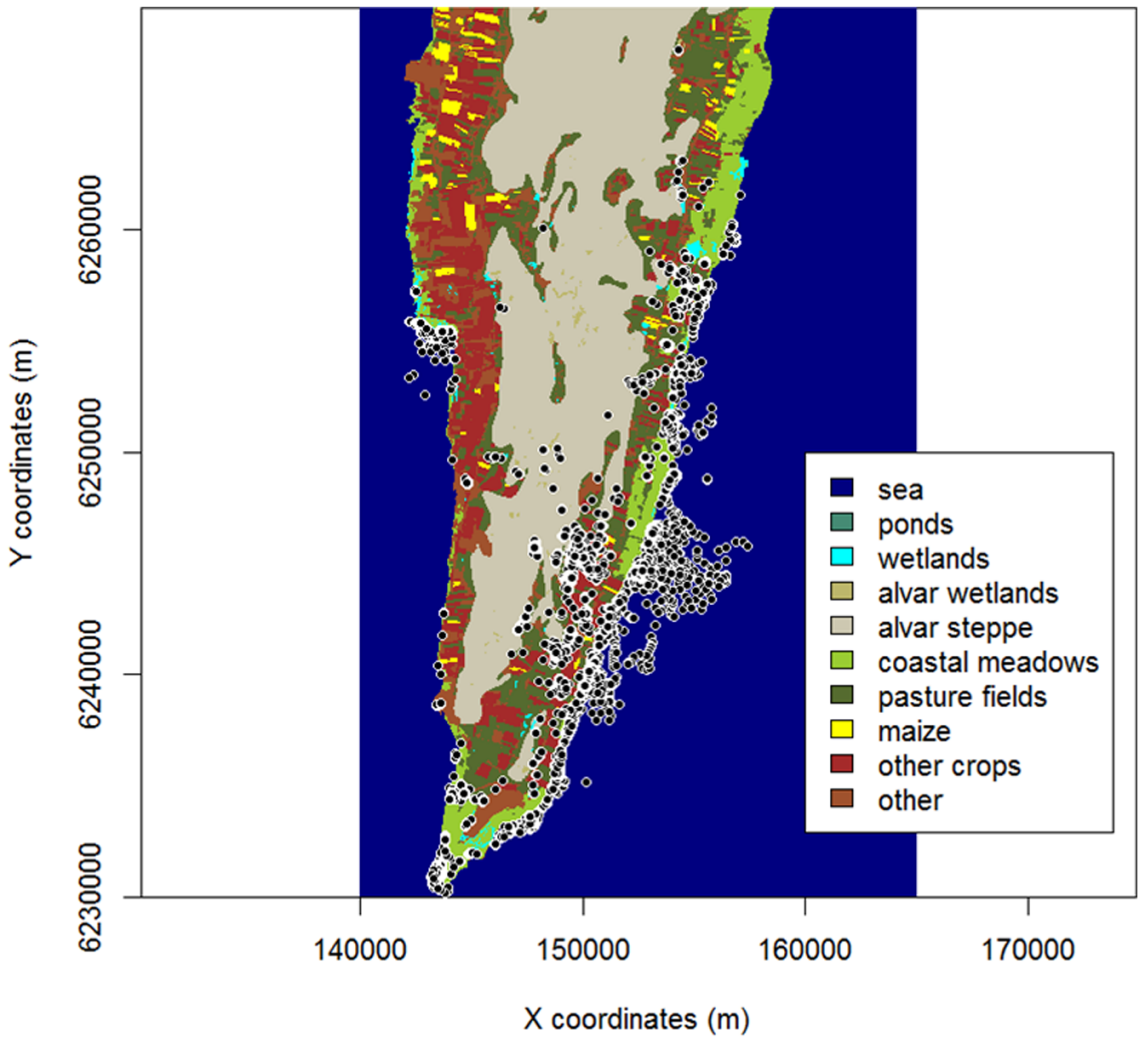
Spatial distribution of mallard GPS fixes (white dots, all individuals pooled) on southern Öland, Sweden, October – December 2010. Fixes from flying birds (speed >4 m/s) are excluded.

A habitat category map was obtained from the Kalmar County Administrative Board. Each data point was assigned a habitat type in order to calculate individual habitat use and habitat selection. The habitat map was rasterized into 10 m square pixels using ArcGis 10 (ESRI 2011) and visualized with the package SMDtools for R software (R Development Core Team 2010). We considered two main habitat categories, *dry* and *wet*, further subdivided into more specific groups. *Wet* habitats were *coast/sea*, *alvar wetland* (generally small, 0.01–0.5 ha wetlands situated in the alvar steppe landscape), *ordinary wetland* (i.e. non-alvar wetlands), *flooded area* (field or alvar in connection to stream or ditch/canal), *wet areas on coastal meadows* (often grazed) and *pond* (man-made). *Dry* habitats consisted mainly of different types of cropland; *pasture*, *maize*, *rapeseed*, *wheat*, *barley*, *rye*, *beans*, *peas*, *strawberries* and *triticale* (Figure 1), but also included a few *other habitats*, such as *farm backyard* and *golf course*.

### Home-ranges and distances between consecutive locations

We investigated the spatial activity of each individual per day (24-hour-period) using two quantitative measures: the 100% Minimum Convex Polygons (hereafter MCP) and the distances between consecutive locations (hereafter Dcl). Days (24 h) were divided into four periods based on expected differences in behavior: *dawn*, defined as the period from first light to sunrise (close to two hours duration); *day*, defined as the period from sunrise to sunset (7–9 hours); *dusk*, defined as the period from sunset to last light (close to two hours duration); and *night*, defined as the period from last light to first light (11–13 hours). MCP is classically used for estimating home-range size [40], although kernel estimators [41] and LoCoH (Local Convex Hull; local kernel associated with a local convex polygon) [42] give better estimates of home-range size by excluding unexplored areas within home-ranges. However, we preferred MCP home-range estimates as they better describe an individual's degree of exploration.

We investigated whether the ducks adopted different searching and exploring activities during *dawn*, *day*, *dusk*, and *night*, using linear mixed models with individual fitted as a random term to account for daily repeated responses of MCP and Dcl for a given individual. Since the number of GPS fixes varied among periods of the day, we tested for a period effect on the MCP while controlling for the number of fixes, using the model *MCP = nloc+per*, where *nloc* is a continuous explanatory variable giving the number of fixes for each period, and *per* is the four-level factor period. The response variables MCP and Dcl were transformed using the Box-Cox transformation[43] to fit normality assumptions. The effect of period was evaluated with the Akaike Information Criterion (AIC) [44] by comparing the models with and without a period effect. All analyses were carried out using the package lme4 for the R software 2.14.

### Habitat selection

Following the approach of Manly *et al*. [45], we investigated patterns of habitat use using selection ratios (*w*). Habitat selection can be defined as preference for a specific environment [46]–[48]. The preference for a given habitat *j*, given by its selection ratio *w_j_*, is calculated as the number of times a Resource Unit (RU  =  patch or pixel) within the habitat *j* is used, divided by the available number of RUs for that given habitat. Under the hypothesis of no particular selection or avoidance, *w_j_* should approach one, which means that the habitat *j* is used in proportion to its availability.

We used Manly's selection ratios for habitat selection design III, which has been developed for studies where each animal is identified and both utilized and available RUs are measured for each individual. We considered all the RUs within an individual's MCP as available RUs, because the study area permits a mallard almost total freedom to roam wherever it chooses. Habitat selection was tested at the individual level using Pearson's Chi-square statistic by comparing the observed selection ratios to the expected selection ratios [45]. Then, for each habitat *j* and animal *i*, we computed the *w_ij_* and associated confidence intervals to identify which habitats that were selected for. All analyses were conducted using the ADEhabitat package [49] for the R software 2.14.

## Results

### General description of daily routines

**Figure 2:**
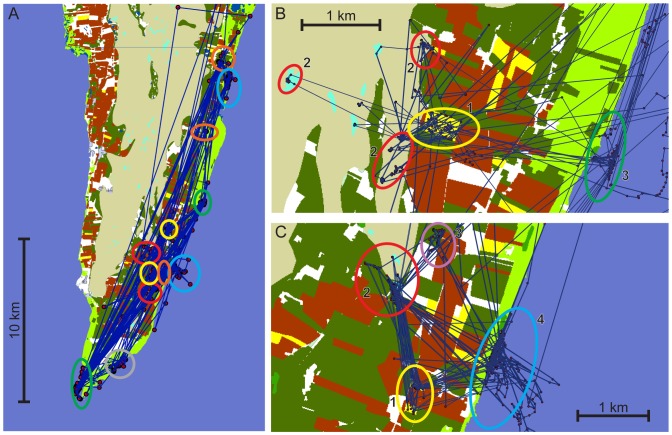
Example of typical mallard movements between frequently used sites on southeast Öland, Sweden, October – December 2010. A (no. 1463): Orange ovals  =  coastal meadows; yellow ovals  =  maize fields; red ovals  =  flooded areas; blue ovals  =  coastal day-roosts (the upper is a sheltered bay, the lower is open water); green ovals  =  coastal reefs used as day-roosts; grey circle  =  duck trap location. B (no. 1426): Yellow oval (1)  =  maize field visited during dawn and dusk; red ovals (2)  =  various small (flooded) wetlands on alvar steppe (the upper one reaching into a maize field), visited at night; green oval (3)  =  coastal reef used as day-roost. C (no. 1440): Yellow oval (1)  =  two maize fields frequently visited, mostly during dawn and dusk; red oval (2)  =  flooded area (stream) visited most nights; light purple oval (3)  =  flooded pasture visited during two consecutive nights (probably for feeding); blue oval (4)  =  most frequented day-roost.

The 16 mallards remained in the study area for on average 31 days (range: 15-38) during their recorded stopover, all of them in the southern part of the island and within 40 km from where they were trapped. Most of them frequented the Ottenby area and the eastern side of the island (Figure 1); only one individual spent most of its time on the western side. Most of the 16 mallards showed a common daily routine that can be described as follows: 1) departure from day-roost 45 minutes after sunset; 2) 30-minute visit in a crop field (often maize); 3) arrival at a small permanent or seasonal wetland on the alvar steppe or close to the coast 45 minutes before complete darkness; 4) departure from “night-area” 45 minutes after first light; 5) 30-minute visit in a crop field (often the same field as the one used the previous evening); 6) arrival at day-roost 45 minutes before sunrise (see Figure 2B and 2C). However, there were many exceptions from this schedule, for example frequent flights between wetlands and crop fields during the night.

### Habitat use

**Figure 3:**
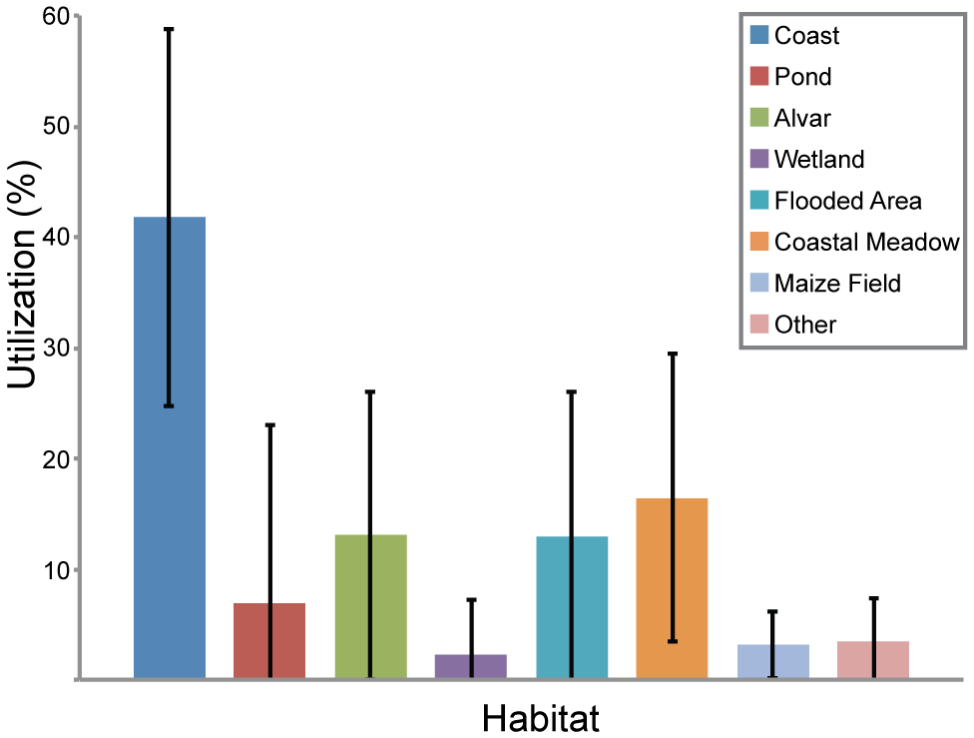
Percentage of time spent by mallards in different habitats (all individuals, with standard deviation bars) on southern Öland, Sweden, October – December 2010.

Fourteen of the 16 ducks spent about half of their total time along the coast (mean 47%, SD = 9.6%), which was the typical habitat for resting during the day. Two individuals used inland ponds as day-roosts instead, in which they spent 31% and 61% of their total time, respectively. For all individuals, when days were split in periods, 96% (SD = 8.0%) of the *day* (sunrise to sunset) was spent at either coast or pond (Table 2). Exclusion of one individual which temporarily used a wetland as its day-roost, increased this percentage to 98% (SD = 2.3). All 16 mallards spent on average 49% (SD = 8.4) at a presumed day-roost locality (Figure 3, Table 3).

**Table 2:** Habitat use time-budgets (in %) from sunrise to sunset for 16 mallards studied on southern Öland, Sweden, October – December 2010.

Individual	Coast	Pond	Alvar (flooded)	Wetland (permanent)	Flooded area (outside alvar)	Coastal meadow	Maize	Other habitat	Number of days followed
1425	29	71	0	0	0	0	0	0	16
1426	98	0	0	0	0	2	0	0	22
1427	98	0	0	0	0	2	0	0	20
1429	97	0	0	0	0	3	0	0	29
1440	99	0	0	0	0	1	0	0	16
1441	97	0	0	0	0	3	0	0	15
1445	2	98	0	0	0	0	0	0	23
1451	96	0	0	0	0	4	0	0	21
1452	97	0	0	0	0	2	0	1	31
1456	97	0	0	0	0	3	0	0	32
1457	92	6	0	0	0	2	0	0	23
1459	67	0	0	32	0	1	0	0	18
1460	99	0	0	0	0	1	0	0	34
1461	72	28	0	0	0	0	0	0	25
1462	90	1	0	0	2	7	0	0	38
1463	99	0	0	0	0	1	0	0	28
Mean all	85	11	0	2	0.1	2	0	0.1	14
StDev	28	29	0	8	0.5	2	0	0.3	6.9

**Table 3:** Total habitat use time-budgets (in %), home-range size (100% MCP; in ha), and number of GPS fixes for 16 mallards studied on southern Öland, Sweden, October – December 2010.

Individual (f) = female (m) = male	Coast	Pond	Alvar (flooded)	Wetland (permanent)	Flooded area (outside alvar)	Coastal meadow	Maize	Other habitat	Home-range size	Number of GPS fixes
1425 (m)	13	31	5	0	37	6	5	3	2176	1374
1426 (m)	40	2	24	0	11	13	8	2	6519	2051
1427 (m)	45	0	37	0	8	1	4	5	6822	1845
1429 (f)	46	0	1	2	0	49	1	0	6031	2622
1440 (f)	44	0	9	0	40	1	6	1	2393	1389
1441 (m)	66	0	9	0	13	12	0	1	348	1330
1445 (f)	1	61	0	0	6	28	0	3	1145	2102
1451 (m)	63	0	2	1	1	19	0	15	1649	1850
1452 (m)	54	0	31	0	4	4	3	4	11932	2797
1456 (m)	42	0	17	0	17	20	3	1	6937	2913
1457 (m)	38	3	10	0	33	10	5	1	11305	2051
1459 (m)	52	0	1	14	0	33	0	0	4752	1571
1460 (m)	51	0	4	0	17	22	4	2	7182	3083
1461 (m)	30	12	32	17	2	0	1	5	4539	2284
1462 (m)	39	2	25	0	6	20	4	4	23857	3390
1463 (m)	46	0	1	0	13	25	6	9	12153	2513
Mean all	42	7	13	2	13	16	3	3	6859	2198
StDev	16	17	13	5	13	13	3	4	5872	640

The remaining 51% (SD = 8.3%) of their time use was mainly spent in freshwater habitats and, to a lesser extent, in crop fields (Figure 3, Table 3).

Most ducks spent a considerable proportion of the dark hours in small (most less than 50 m in diameter), permanent or seasonal, natural wetlands on the alvar steppe, 3–5 km from the coast. On average, the 16 ducks spent 23% (SD = 23.3%) of their night-time, and 13% (SD = 12.8%) of their total time, in such wetlands (Figure 3, Table 3, Table 4). The variation was large, however. For example, 5 mallards spent on average 54% (SD = 10.6%) of their nights at alvar wetlands. Also seasonally flooded areas (connected to small streams and located up to 3 km from the coast) were used intensively, mainly at night. Three mallards spent 58%, 65% and 72%, respectively, of their night-time in such flooded areas (Table 4). The third major freshwater habitat used by the mallards was flooded coastal meadows, where 6 of the birds spent >45% of their time away from day-roosts (mean  = 63%, SD = 18.7%), and 8 ducks spent >25% of their night-time (mean  = 46%, SD = 18.3%) (Table 4). Only 2 ducks spent a considerable proportion (29% and 30%, respectively) of their time away from day-roosts in permanent wetlands outside the alvar. Altogether, the four categories of shallow freshwater habitats were used on average 87% (SD = 9.6%) of the total time away from day-roosts (Figure 3, Table 3), and 76% (SD = 15.8%) of the total night-time (Table 4).

**Table 4:** Habitat use time-budgets (in %) from last light to first light for 16 mallards studied on southern Öland, Sweden, October – December 2010.

Individual	Coast	Pond	Alvar (flooded)	Wetland (permanent)	Flooded area (outside alvar)	Coastal meadow	Maize	Other habitat	Number of days followed
1425	1	6	8	0	65	10	5	5	16
1426	1	3	43	0	19	22	10	2	22
1427	10	0	66	0	14	1	5	5	20
1429	9	0	2	3	0	83	2	0	29
1440	6	0	15	0	72	0	6	0	16
1441	42	0	16	0	23	17	0	1	15
1445	0	37	0	0	10	51	0	2	23
1451	38	0	4	3	1	31	0	24	21
1452	22	0	59	0	7	5	3	5	31
1456	3	0	31	0	31	33	2	1	32
1457	1	1	18	0	58	15	5	0	23
1459	40	0	1	0	0	59	0	0	18
1460	18	0	7	0	30	37	5	2	34
1461	0	0	60	31	3	0	1	5	25
1462	6	3	43	0	9	29	5	5	38
1463	10	0	1	1	22	43	8	14	28
Mean all	13	3	23	2	23	27	4	5	14
StDev	15	9	23	8	23	24	3	6	6.9

Twelve mallards (75%) visited maize fields, spending on average 4% (SD = 2.0%) of their total time in that habitat (Figure 3, Table 3), and 12% (SD = 5.8%) of the twilight hours (first light to sunrise and sunset to last light). Pastures were visited by all individuals, but only 5 ducks spent more than a total of 3 hours in this habitat; on average 18 hours (SD = 13.9 h) or 3.1% (SD = 3.4%) of their total time-budgets. However, almost half (42%) of the data points ascribed to pastures referred to one mallard visiting a specific pasture that was most probably flooded. The presence of rain water pools probably explains why two mallards spent 6% and 5%, respectively, of their time in bean fields, whereas the rest spent no time at all in this habitat. Some mallards also visited harvested fields with residual rapeseed, wheat or barley, but such fields never made up >5% of an individual's total habitat use.

### Home-range and distance analysis

The mallards in our study often flew short (<1 km) or fairly short (3–5 km) distances between foraging areas and day-roosts. However, some ducks regularly travelled up to 26 km between Ottenby and selected foraging sites. Hence, some used a limited area whereas others explored large areas. The 100% MCP home-ranges for the 16 mallards differed considerably in extent, ranging from 348 to 23,857 ha (mean  = 6,859 ha; SD = 5,872 ha; Table 3). As expected, individual MCP size was positively related to the number of fixes recorded (linear model after log-transformation of the MCP size, slope = 0.001±3*10e-4, student t-test *p*<0.01). We therefore controlled for the number of fixes when analysing the effects of the different periods of the day (24 hours) on MCP size.

**Figure 4:**
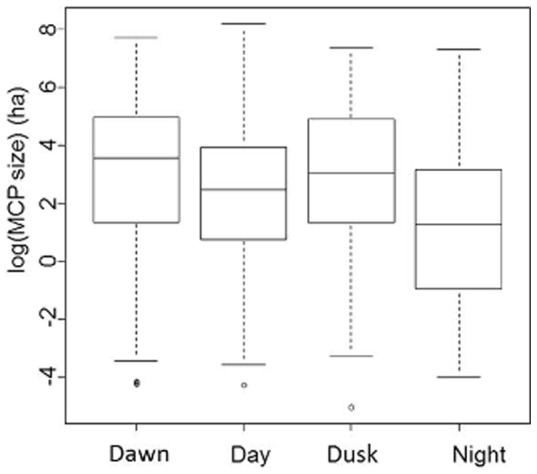
Home-range size (MCP in ha on a log scale) of mallards on southern Öland, Sweden, October – December 2010, during different periods of the day; *dawn*  =  first light to sunrise (duration close to two hours), *day*  =  sunrise to sunset (7–9 hours), *dusk*  =  sunset to last light (duration close to two hours), and *night*  =  last light to first light (11–13 hours).

Although the time periods *dawn* and *dusk* each make up less than 10% of the total 24-hour-period, the areas explored during these time periods were significantly larger than during the time periods *day* and *night* (Figure 4, models *MCP = nloc+per vs MCP = nloc,* AIC = 7,066 *vs* 7,081).

**Figure 5:**
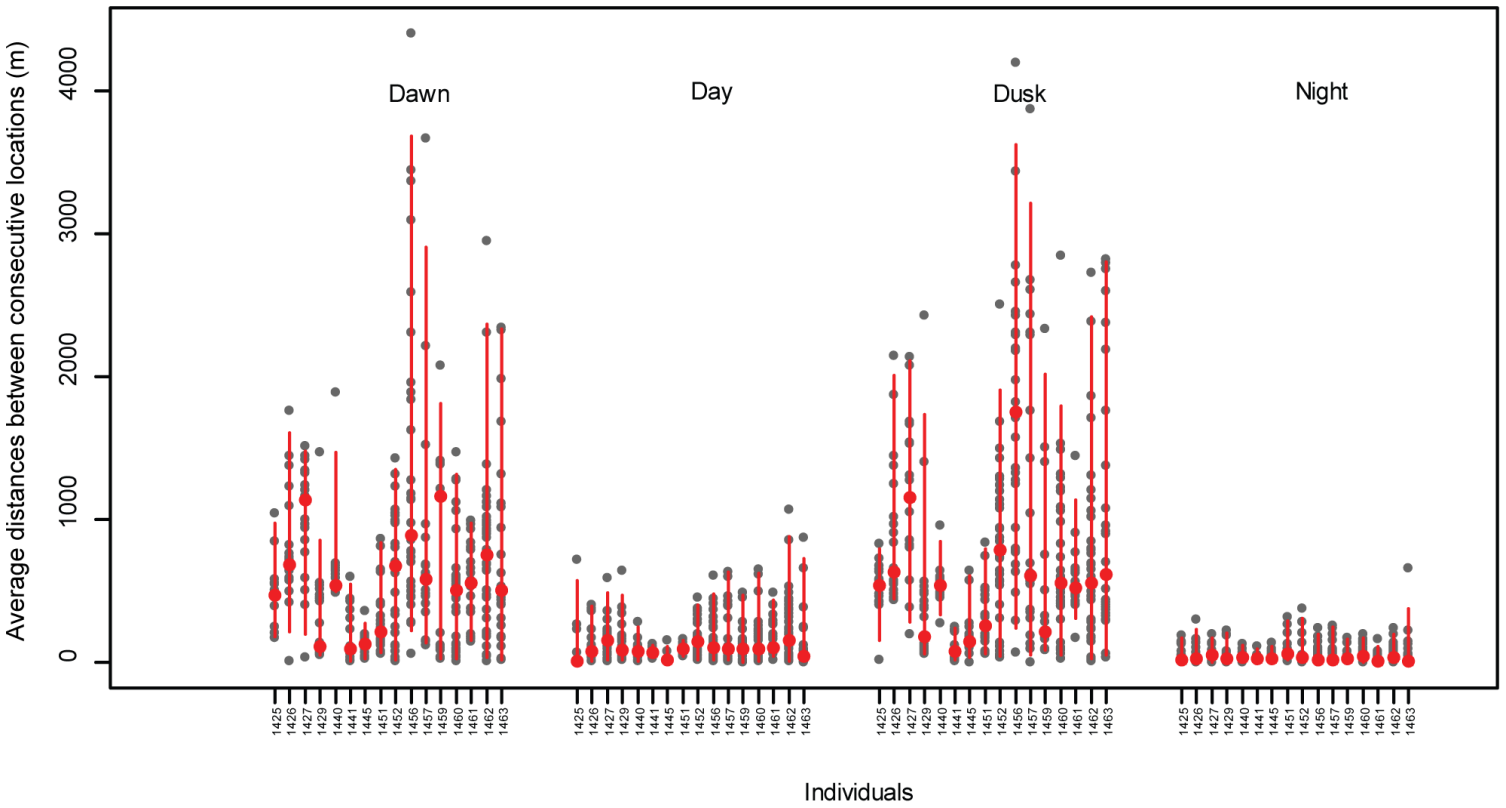
Distance between consecutive locations for 16 mallards on southern Öland, Sweden, October – December 2010, during different periods of the day; *dawn*  =  first light to sunrise (close to two hours duration), *day*  =  sunrise to sunset (7–9 hours), *dusk*  =  sunset to last light (close to two hours duration), and *night*  =  last light to first light (11–13 hours).

Distances between consecutive locations during different periods of the day revealed an even clearer period effect (Figure 5, models *Dcl = per vs Dcl = constant*, AIC = 107,652 *vs* 110,960). Overall, ducks moved over greater distances between fixes during *dawn* and *dusk* (average model predictions: 37.54 m, 95%CI [35.50, 39.72]; 56.54 m, 95%CI [53.31, 60.00], at *dawn* and *dusk*, respectively), whereas they were more stationary during *day* and *night* (average model predictions: 12.13 m, 95%CI [11.56, 12.72]; 7.53 m, 95%CI [7.20, 7.88], during *day* and *night*, respectively).

### Habitat selection analysis

Habitat selection at the home-range level showed a strong preference for ponds and alvar wetlands in all ducks (Table 5; χ^2^ = 3,276; *df* = 96; *p*<<0.001). Despite the fact that ponds and alvar wetlands are relatively scarce habitat types on Öland, they were highly preferred by mallards in our dataset. This preference outweighed preference for more common habitat types to the point where it became difficult to determine whether these individuals also selected habitats other than ponds and alvar wetlands. In order to further explore selection, all wet habitats were pooled together. This resulted in a clear selection for maize fields (Table 6, test of overall departure from non-preference: χ^2^ = 18,843; *df* = 73; *p*<<0.001).

**Table 5:** Selection ratios (*w_i_*) at the home-range level, averaged over 16 mallards studied on southern Öland, Sweden, October – December 2010, with respective 95% confidence interval (CI) lower and upper limits.

Habitat	*w_i_*	CI lower	CI upper
Ponds	143.82	−7.96	295.61
Alvar wetlands	29.99	12.57	47.41
Other wetlands	6.11	−2.91	15.13
Maize fields	2.95	1.54	4.36
Coast and sea	1.49	0.80	2.19
Coastal meadows	1.20	0.44	1.95
Alvar steppe	0.77	0.04	1.50
Pasture fields	0.34	0.15	0.54
Other crops	0.17	0.02	0.32
Other habitat	0.08	0.03	0.13

The table shows the selection ratios when considering the habitat as a ten-level factor (wet habitats are divided in subcategories). The habitats are ordered from the most (top) to the least preferred habitat (bottom).

**Table 6:** Selection ratios (*w_i_*) at the home-range level, averaged over 16 mallards studied on southern Öland, Sweden, October – December 2010, with respective 95% confidence interval (CI) lower and upper limits.

Habitat	*w_i_*	CI lower	CI upper
Maize fields	2.95	1.63	4.28
Coast/wetlands	1.90	1.39	2.42
Alvar steppe	0.77	0.08	1.45
Pasture fields	0.34	0.16	0.53
Other crops	0.17	0.03	0.31
Other habitat	0.08	0.04	0.13

The table shows the selection ratios when considering the habitat as a six-level factor (all wet habitats are pooled together). The habitats are ordered from the most (top) to the least preferred habitat (bottom).

**Figure 6:**
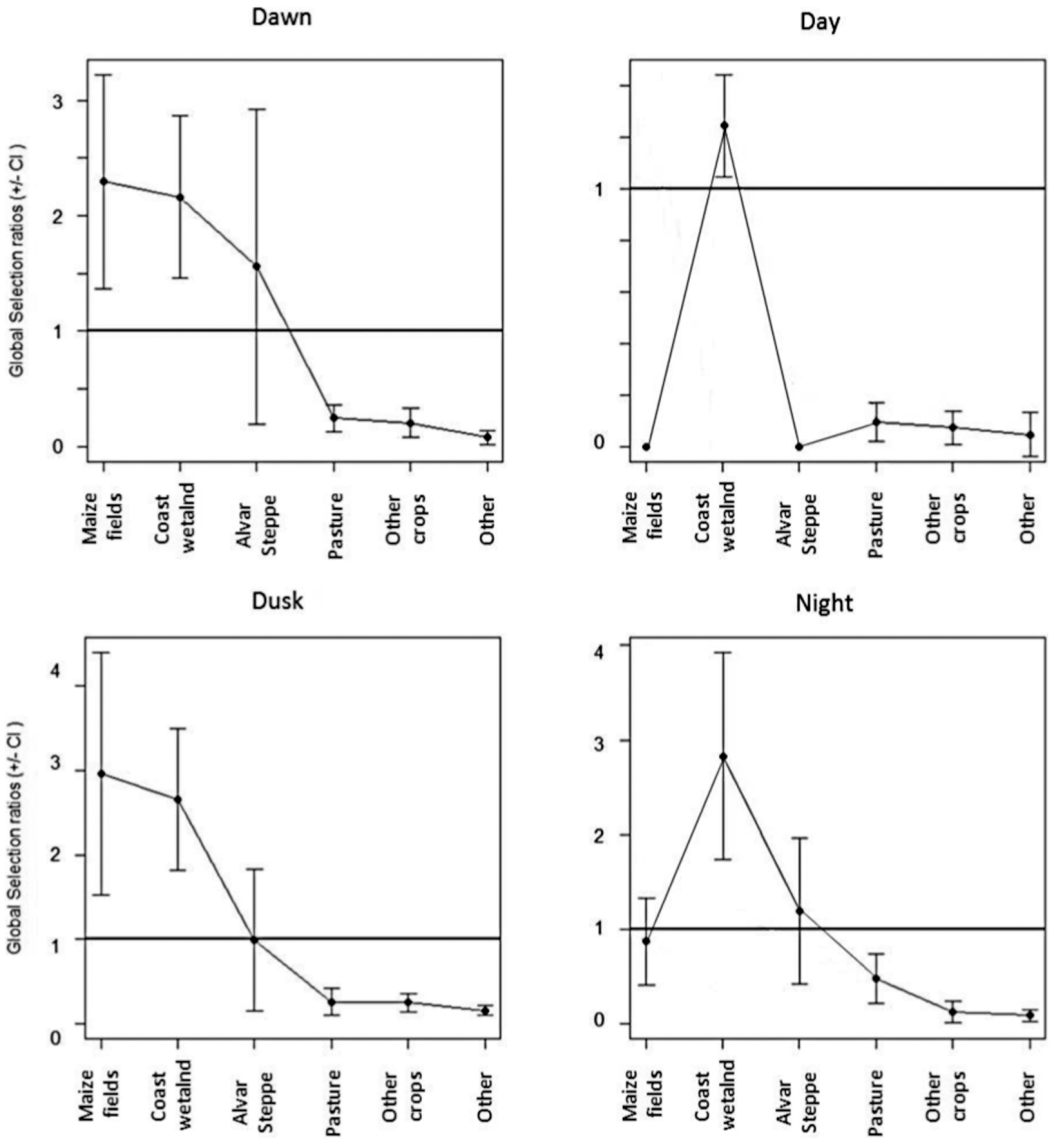
Manly et al.'s selection ratios (*w_ij_*±95% CI) for each period of the day. Habitat is considered as a six level factor, i.e. all wetland habitats have been pooled.

Investigating the pattern of habitat selection for each of the four periods of the day, we found a clear shift in habitat preference from the periods *dawn* and *dusk* compared to *day* and *night* (Figure 6). There was a clear preference for maize fields during *dawn* and *dusk* (tests of overall departure from non-preference, χ^2^ = 1,821; *df* = 55; *p*<<0.001 and χ^2^ = 1,922; *df* = 64; *p*<<0.001, respectively), while wetlands were preferentially selected during *night* and *day* (test of overall departure from non-preference: χ^2^ = 13,096; *df* = 65; *p*<<0.001 and χ^2^ = 2,695; *df* = 19; *p*<<0.001, respectively).

## Discussion

### Habitat use

Our study demonstrates that autumn-staging mallards have a clear diel pattern comprising major shifts in activity and habitat preference. The tracked mallards spent their daylight hours mostly at coastal localities, strikingly often at reefs in protected areas (often in nature reserves), and visited fields and inland wetlands during the night. Flight activity was highest at *dawn* and *dusk* (Figure 4, Figure 5), when ducks moved between day-roosts and foraging areas, which is in accordance with previous studies [32], [34]. Although some individuals were exceptions from this behaviour, and ducks often made additional flights, by and large mallard diel movements were similar among individuals and predictable in time. Some of the ducks in this study displayed fixed behavioural patterns, whereby they moved between the same habitat locations, at similar times, for many consecutive days. Moreover, we found that these predictable movements were often performed by pairs or groups of individuals evidently moving together.

Nocturnal foraging is generally attributed to more profitable foraging conditions (e.g. availability of nocturnally active prey) or lower predation risk [25]. Diurnal predators, such as raptors, are usually abundant in the study area in autumn, and Öland holds a vital population of red fox (*Vulpes vulpes*). Duck hunters are also present on Öland, but few in number. Since the hunting pressure is low (according to hunting reports and personal observations), it is unlikely to affect the foraging patterns of our study ducks to any large extent.

Day-roost and night-foraging patterns in winter have been described for the mallard, as well as for other dabbling ducks [28], [30], [34], [50], but the amount of night-foraging may differ within and between species [51]. In western France, wintering ducks spent 37–60% of their time foraging; on average 16% of daylight hours and 85% of the night [33]. The Eurasian teal has been shown to forage 88% of the total time on a daily basis in February – August [10], but only 46% in winter [26].

We found that the mallards in our study spent approximately equal time in “foraging” versus “resting” habitats (Figure 3, Table 2–4). This is an important insight, as we are not aware of any other study providing total time-budgets for habitat use of dabbling ducks during stopover. Admittedly, we cannot be certain that the mallards actually foraged in the shallow freshwater habitats that were visited mostly at night, as they may have filled their crops when visiting crop fields at dusk. However, it seems unlikely that they did not forage in the freshwater habitats, as it would have been equally possible, and probably much safer, to return to the coast to digest the crop contents. The obvious pattern of distinctly different habitat use in day and night also indicates that the mallards used freshwater habitats for foraging (and drinking).

There was considerable variation in how much time individuals spent at day-roosts versus foraging areas (Table 3). This could be explained by the possibility that some day-roosts offered foraging opportunities, thereby reducing the need for foraging at night, but may also reflect different needs related to individual body condition. Although some foraging probably occurred at the day-roost localities, it was likely limited as the reefs and bays do not offer good foraging habitats (brackish and fairly open water with little vegetation to feed on for a dabbling duck). Our data suggest that foraging times were approximately the same as for wintering ducks in France [26], [33].

### Home-range and distance of movements

The home-ranges observed in our study are some of the largest ever documented for mallards, albeit with high standard deviations (Table 3, Table 7). This was mainly due to the relatively long travel distances performed by some individuals between day-roosts and night-time foraging areas. Observed distances between consecutive locations verify that longer movements were undertaken during *dawn* and *dusk*, while mainly shorter distances were covered during *day* and *night* (Figure 5).

**Table 7:** Previously published home-range sizes for mallards in comparison to the present study.

Study/Author	Country (state)	Season	Home-range size (in ha)	Standard deviation	Number of ducks
This study	Sweden	autumn	6859	5872	16
Jorde et al. 1984	USA (Nebraska)	winter	3243.7**	638.7	14
Legagneux et al. 2009	France	winter	1257.3	1915.1	15
Legagneux et al. 2009	France	winter	496.2	861.3	8
Legagneux et al. 2009	France	winter	326.6	162.7	8
Legagneux et al. 2009	France	winter	221.0	242.8	7
Legagneux et al. 2009	France	winter	184.7	147.7	7
Sauter et al. 2012	Switzerland	winter	min 16* max 6367*	not available	33
Dwyer et al. 1979	USA (North Dakota)	breeding (females)	467.8	158.6	6
Kirby et al. 1985	USA (Minnesota)	Breeding (females)	343.8	391.4	5
Gilmer et al. 1975	USA (Minnesota)	Breeding (males)	240	not available	12
Gilmer et al. 1975	USA (Minnesota)	Breeding (females)	210	not available	12

(Further examples exist, especially for breeding birds, but our aim here is to show a representative variation.).

*Mean values not published.

**This mean value does not include foraging areas.

The reefs where most ducks spent the days were fairly safe from predators, while the fields/wetlands where the ducks spent the nights were obviously attractive in terms of foraging opportunities. This suggests that the benefits obtained from these habitats outweighed the costs (such as energy expenditure and predation risk) incurred by moving between them. In areas where similar studies have been performed in winter, extensive wetlands make the ducks less prone to change foraging sites, and travelling distances are usually shorter. Wintering dabbling ducks usually move less than 25 km on a daily basis, based on studies of mallard [34], [52], [53] and pintail [54]. Home-ranges for wintering mallards seldom exceed 5,000 ha [34], [55], [56] and for breeding birds they are usually much smaller [57]–[60] (Table 7). It could be that birds learn to minimize home-range size more effectively in winter, compared to the shorter stopover periods in spring and autumn, respectively. Also, the need to minimize energy loss is usually higher in winter, as thermoregulation can be costly in cold temperatures.

The 3 females in our study had a much smaller average home-range size (3,190 ha; SD = 2,539 ha) than the 13 males (7,705 ha; SD = 6,105 ha). Our low sample size of females does not permit any conclusions to be drawn, although sex (and age) could possibly affect home-range size. Jorde *et al*. (1984) argued that young birds may need to explore larger areas due to inexperience [55]. The latter study also reported larger home-ranges for males than females, but with a rather small sample size (winter study; 14 mallards divided in 4 age/sex groups).

### Habitat selection

The importance of alvar wetlands as a stopover habitat for mallards was previously unknown, although inland wetlands are generally considered very important to dabbling ducks [61], [62]. This finding has important implications beyond mallard autecology since dabbling ducks are considered to be main vectors of propagules (e.g. eggs, seeds, oogones) of aquatic organisms between isolated wetlands [63]. This can be considered an important ecosystem service [64], but possibly also a concern when it comes to spread of invasive species [65].

A large proportion of coastal southern Sweden is heavily farmed, but ducks have seldom been observed foraging in fields. However, our data show that they frequently do so. In the study area, maize fields are harvested at the end of the summer, leaving abundant residual maize for mallards to forage. Maize seems to be the most attractive crop to our staging mallards (Figure 6, Table 3, Table 4), a pattern also observed in other areas [53]. This is despite the fact that the birds in our study (all juveniles) most likely have never encountered maize before (it is not grown closer to the main breeding grounds of mallards farther northeast in Sweden, Finland, and Russia). This might imply a benefit for the mallard, being a generalist more than a specialist feeder. Furthermore, we found evidence in our data that some of the mallards moved in groups between fixed habitat locations. It is quite possible that the juveniles we tracked travelled alongside adult individuals who had previously stopped-over on Öland during previous migrations, and learnt to utilize grain fields from individuals with prior knowledge of the landscape.

The proportion of maize fields compared to other crops has increased in the study area in recent years. Until 2005, <1% of the farmland on southern Öland (the municipality of Mörbylånga) was used for maize, whereas in the last few years it has been grown to encompass >6% of the agricultural land. At the national level, maize fields cover only 0.6% of the total agricultural area used for crops in Sweden, but have increased by >400% from 2001 (3,161 ha) to 2012 (16,482 ha). This change of farming preferences could have an impact on the stopover ecology of mallards travelling through Sweden, as has been observed for other bird species. Several species of geese have recently become much more numerous in response to increasing availability of autumn-sown crops and sugar beet spill (due to machine harvesting) in Sweden and beyond [66]. The pink-footed goose (*Anser brachyrhynchus*) population on Svalbard has increased dramatically, mainly due to improved feeding conditions at stopover and wintering sites [67].

The frequent but usually very brief visits (most often 30 minutes or less) in maize fields observed in this study, could possibly be because most fields on Öland are located between the coast and the alvar steppe, i.e. between major foraging/roosting areas. But the geographically convenient location is probably not the main reason for the popularity of maize fields. For example, some ducks made several visits during a single night, by repeated flights between a wetland and a maize field.

We suggest that predation pressure, being a strong determinant of foraging behaviour [10], [68]–[70], is the most likely reason for the brief visits in maize fields. The risk of predation could also explain why the ducks preferred to visit maize fields in twilight, when diurnal raptors are usually not active and it is still possible to discern nocturnal predators (e.g. fox) by sight. According to optimal foraging theory, even a highly beneficial habitat or behaviour should only be used when the risk of predation is relatively low [71]–[73]. It could be that the ducks quickly fill up their crop and then leave for safer grounds.
